# Alliance members’ roles in collective field-building: an assessment of leadership and championship within the Population Health Intervention Research Initiative for Canada

**DOI:** 10.1186/s12961-017-0265-x

**Published:** 2017-12-06

**Authors:** Erica Di Ruggiero, Natalie Kishchuk, Sarah Viehbeck, Nancy Edwards, Kerry Robinson, Barbara Riley, Heather Smith Fowler

**Affiliations:** 1 Dalla Lana School of Public Health, University of Toronto, 155 College Street, Suite 408, Toronto, Ontario M5T 3M7 Canada; 2 Program Evaluation and Beyond, Université de Montréal, Montréal, Canada; 3 School of Public Health and Health Systems, University of Waterloo, Ottawa, Canada; 4 Interdisciplinary School of Health Sciences, University of Ottawa, Ottawa, Canada; 5 School of Nursing, University of Ottawa, Ottawa, Canada; 6 Public Health Agency of Canada, Ottawa, Canada; 7 Propel Centre for Population Health Impact, University of Waterloo, Waterloo, Canada; 8 Social Research and Demonstration Corporation, Ottawa, Canada

**Keywords:** Field-building, Alliance, Population health intervention research, Research capacity-building

## Abstract

**Background:**

The Population Health Intervention Research Initiative for Canada (PHIRIC) is a multi-stakeholder alliance founded in 2006 to advance population health intervention research (PHIR). PHIRIC aimed to strengthen Canada’s capacity to conduct and use such research to inform policy and practice to improve the public’s health by building PHIR as a field of research. In 2014, an evaluative study of PHIRIC at organisational and system levels was conducted, guided by a field-building and collaborative action perspective.

**Methods:**

The study involved 17 qualitative key informant interviews with 21 current and former PHIRIC Planning Committee and Working Group members. The interviews examined how individuals and organisations were acting as champions and exerting leadership in building the field of PHIR.

**Results:**

Founding PHIRIC organisational members have been championing PHIR at organisational and system levels. While the PHIR field has progressed in terms of enhanced funding, legitimacy, profile and capacity, some members and organisations faced constraints and challenges acting as leaders and champions in their respective environments. Expectations about the future of PHIRIC and field-building of PHIR were mixed, where longer-term and founding members of PHIRIC expressed more optimism than recent members. All agreed on the need for incorporating perspectives of decision-makers into PHIR directions and initiatives.

**Conclusions:**

The findings contribute to understanding alliance members’ roles in leadership and championship for field-building more generally, and for population health and PHIR specifically. Building this field requires multi-level efforts, collaborative action and distributed leadership to create the necessary conditions for PHIRIC members to both benefit from and contribute to advancing PHIR as a field. Lessons from this 'made in Canada' model may be of interest to other countries regarding the structures needed for PHIR field-building.

**Electronic supplementary material:**

The online version of this article (10.1186/s12961-017-0265-x) contains supplementary material, which is available to authorized users.

## Background

The Population Health Intervention Research Initiative for Canada (PHIRIC) was launched in 2006 as a 10-year, multi-stakeholder initiative “*to advance population health intervention research (PHIR) and strengthen Canada’s capacity to fund and use such research to inform policy and practice*”^1^ [1]. PHIRIC defines PHIR as “*the use of scientific methods to produce knowledge about policies, programs, and events that have the potential to impact health at the population level*” [2, 3]. PHIRIC was designed to place greater emphasis on the study of population health interventions in a field primarily characterised by descriptive, correlational and non-experimental research, and a relative dearth of studies examining the implementation and impact of program, policy and practice changes on population health outcomes [4, 5]. PHIRIC aimed to increase opportunities for graduate and postgraduate training in PHIR. It further sought to redress uneven success rates in granting competitions, possibly stemming from poor application quality, lack of appropriate peer-review criteria, and/or lack of reviewer familiarity with the specific nature and complexity of population health intervention studies [4–7].

PHIRIC’s strategic objectives are to:

- advance the science of PHIR;
- strengthen Canada’s capacity to conduct and use relevant PHIR for policy and practice;
- enhance Canada’s contribution to the global knowledge base on population health interventions through continuous learning and international collaborations; and
- champion PHIR and enhance its profile and usefulness.

PHIRIC’s members are individuals and organisations from academia, government, research funding and non-governmental organisations [8]. The founding members developed an initial shared vision at the 2006 launch, and then formed an alliance based on shared interest in building capacity for PHIR as a focus area within public and population health research. Membership was expanded to include others from research, policy and practice sectors who could provide individual and organisational leadership to develop and achieve strategic objectives. PHIRIC is currently governed by a Planning Committee made up of representatives from across these sectors. Some members represent organisations (e.g. the Public Health Agency of Canada, the Canadian Population Health Initiative of the Canadian Institute for Health Information, the Canadian Institutes of Health Research – Institute of Population and Public Health (CIHR-IPPH)), while others participate as individuals (primarily academics but also eminent retirees of former member organisations). Table 1 outlines the committee’s membership and changes in membership over time. To accomplish its collective and evolving work plan, PHIRIC created four Working Groups between 2008 and 2010, each of which was established to advance PHIR in a specific domain, namely Training, Communications, Peer Review Guidelines and Evaluation [9]. From 2006 to 2016, the CIHR-IPPH served as the initiative’s scientific and knowledge exchange secretariat.

**Table 1:** PHIRIC Planning Committee Members

Founding Organizational Members (as of 2006)	Members who joined as of 2007
Canadian Institutes of Health Research-Institute of Population and Public Health	Social Research and Demonstration Corporation (since 2007)
Canadian Institutes of Health Research-Institute of Nutrition, Metabolism and Diabetes	Institut national de santé publique du Québec (since 2007)
Canadian Institute for Health Information-Canadian Population Health Initiative	Provincial Health Services Authority (PHSA) (joined in 2007; left in 2010)
Chronic Disease Prevention Alliance of Canada (CDPAC) (until 2013)	Canadian Council on Learning (CCL) (as of 2007; left in 2010)
Propel Centre for Population Health Impact, University of Waterloo	
Public Health Agency of Canada	

Organizations were represented by at least one but often two representatives in the case of larger organizations (CIHR, PHAC). All founding organizations remained engaged as organizational representatives over the time period with one exception (CDPAC). In some cases, the representative(s) from each organization changed or the individual left the organization but remained engaged and so the connection to the organization was lost (PHSA, CCL, and CDPAC). In addition to organizational representatives, individual experts from research and policy communities were engaged during the time period. Some also served as either a member or co-chair of the initiative, providing an explicit link to the CIHR-IPPH advisory board at the time

After some years of existence, PHIRIC’s Planning Committee undertook an evaluative study of its members’ individual and collective effectiveness in acting as leaders and champions of PHIR at organisational and system levels within and beyond member organisations. This article reports on that study, using frameworks and concepts from the literatures on field-building and collaborative action (e.g. strategic alliances, collective impact, distributed leadership) to elucidate the strategies PHIRIC members have used to achieve outcomes for PHIR.

### Field-building

PHIRIC’s approach to strengthening PHIR capacity in Canada can be situated within the general framework of field-building. In contrast to individual and organisational capacity-building, the field-building framework posits that generating awareness of and support for a new field requires concerted multi-level action to ensure individual and organisational capacities are identified and nurtured in supportive environments. This concerted action should simultaneously involve the development of standards and exemplars [10, 11], a knowledge base of credible, policy-oriented and action-oriented evidence that synthesises conclusions across multiple studies [10, 12], influential scientific leadership by a core cadre [10], broad-based support among key influencers [12], and organised funding streams [10, 12, 13].

Similarly, field-building in applied research requires multiple levels of activity to support the funding, conduct and use of research in a given area [14, 15]. For example, training programmes are required so more researchers acquire the requisite competencies for the new field [10–12, 16, 17]. Team-level capacity-building is needed to achieve sufficient critical mass within research institutions and policy and program organisations [11, 16], to attract, integrate and mentor new researchers [12], and to strengthen the performance of those already in the field. Supportive environments are needed to recognise and value this research in peer-reviewed granting and publication systems supporting integration of research questions into existing policy and programme efforts [12, 15]. Mechanisms and fora for “*purposeful socialization*” ([11], p. 236) are needed where researchers and policy-makers can communicate about each other’s work, and identify opportunities to collaborate and develop networks [10] to connect intellectually isolated groups and individuals [10]. Finally, field-building in research that aspires to influence policy, programme and practice decision-making requires the identification and bidirectional [16], non-hierarchical [10, 16] engagement of committed practice and policy communities, ready to be involved in shaping, commissioning, supporting, conducting and applying research findings in their organisations [12, 16].

### Collaborative action and strategic alliances

The literature on collaborative action suggests ways that leadership and championship in collective field-building for PHIR and other research fields could be exercised by individuals, organisations and systems. With members from different sectors united by a common agenda, PHIRIC has described itself as a “*loosely-structured alliance*” ([18], p. 5). Strategic alliances are “*intentional, inter-organizational collaboratives created to benefit the partners and ultimately the stakeholders that they serve*” [19]. Among their characteristics, shared or transferred decision-making power, and hence shared leadership, is crucial to achieve benefits [20]. Shared leadership to build PHIR requires ongoing commitment from individuals working within and across organisations to recognise and seize opportunities, open pathways, navigate and buffer resistance, stave off competing resource demands, and harness support and resources in pursuit of the common goal (i.e. PHIR) [21]. However, field-builders are often engaged as individuals rather than organisational or system actors, with an accordingly limited sphere of influence [10]. Understanding the effectiveness of distributed leadership in PHIRIC requires analysis of the actions of networked leaders at multiple levels – individual, organiaational and systemic [22].

Collective impact is another relevant perspective related to collaborative action. It has been advanced to address a need for shared leadership and responsibility to achieve meaningful population-level change in social conditions [23]. Collective impact initiatives are defined as “*long-term commitments by a group of important actors from different sectors to a common agenda for solving a specific social problem*” ([24], p. 36). This movement has grown out of recognition that successive small-scale demonstration projects, while producing results that are potentially applicable at larger scales such as entire jurisdictions, communities or sectors, rarely achieve widespread and sustained adoption and application without deliberate and appropriately resourced efforts across multiple organisations [25]. Collective impact can provide a useful frame for understanding collective field-building.

#### Study purpose

In the context of field-building for PHIR, PHIRIC members are intended to act as leaders and organisational champions of PHIR, within and across their organisations through a distributed leadership approach [26]. The present study examined to what extent and how current and former PHIRIC members were engaged as:

- individuals, championing PHIR within their organisations by taking on internal leadership roles to gain support and resources for the overall field of PHIR and for the specific role of PHIRIC as a field-developing instrument;
- representatives of their organisations (where relevant), taking on external leadership roles to gain support and resources for PHIR initiatives, and for PHIRIC as a legitimate actor in population health research capacity development (Table 1); and
- members of PHIRIC, assuming collective responsibility to act as leaders and champions to advance the field of PHIR in Canada.

## Methods

Using a participatory evaluation approach, PHIRIC’s Evaluation Working Group (EWG)^2^ provided leadership for this evaluative study on behalf of the PHIRIC Planning Committee. Consistent with its Terms of Reference, the EWG considered this study an opportunity to inform PHIRIC’s future directions and plans.

### Study participants

All current and former PHIRIC members were categorised by membership status (current or former), level (individual or organisational), and sector (research, policy or research funding). Potential interviewees were purposively selected to provide balance across these categories. Within organizations, the individual who was most closely involved with PHIRIC was selected to ensure representativeness. Former members included founding members who had left for reasons unrelated to PHIRIC (e.g., retirement, change of job), and organizations that had voluntarily pulled back from membership or active participation, between one and four years prior to the study. EWG members were included in the sample.

All of those organisations and individuals contacted for interviews agreed to participate. Seventeen qualitative semi-structured key informant interviews were conducted. Two interviews involved two participants and one interview included three participants, for a total of 21 individuals. Ten of those interviewed were in academic positions or in research or evaluation organisations, six were from research funding agencies, and five were employees of policy and practice organisations. Eleven interviewees (of 17) stated they were founding members or were involved from the beginning. Of these, six were organisational representatives.

### Interview guide

The exploratory, semi-structured interview guide (Additional file 1) was developed iteratively by the EWG, based on the study aims. There were three broad domains of inquiry:

1. respondents’ roles in leading and championing change to support the development of population health intervention research, within their organisation, within PHIRIC, and within the population and public health system more broadly – and how that role was exercised;
2. the nature and level of influence exerted by PHIRIC members on developing population health intervention research; and
3. what PHIRIC could do to further support population health intervention research within members’ organisations, and more broadly.

Interviewees were asked to specify whether they were speaking as an individual or a representative of their organisation. The interview guide questions were adjusted according to their specification.

### Data collection

Interviews were conducted by an independent evaluation consultant (NK) experienced in public health research and evaluation, not directly involved in the PHIRIC Planning Committee but familiar with the field of PHIR. The interviews were conducted by telephone in either French or English during January and February 2014, and lasted an average of 45 minutes.

### Data treatment

The interviewer took detailed notes during the interviews. Using voice recognition software, near-verbatim responses to interview questions were dictated immediately after interviews. The data were organised into three matrices corresponding to the three inquiry domains, with interview questions as columns, interviewees as rows and near-verbatim content as cell entries. Interviews were not audio-recorded. These data were then subject to an initial content analysis by the interviewer, who then proceeded to identify emerging themes within each column/question [27]. Data from all interviewees were weighted equally in the analysis.

### Data analysis

Since understanding is limited on effective leadership and championship in field-building, the analytic approach for this study was essentially inductive. Two participatory analysis sessions were held with the EWG to develop and refine emergent themes. These sessions resulted in a decision to further stratify respondent types to take into account their different perspectives as individual or organisational representatives, and to distinguish longer-term PHIRIC members (including founding members who were still engaged, even if peripherally) from newer and former members. Matrices were then used to compare responses to questions among these subgroups. These emergent results were presented to the full PHIRIC Planning Committee to develop consensus on final results and to discuss their implications.

## Results

### Leadership and championship of change

The interviews indicated that, at the level of their own organisations, PHIRIC members have been building the field of PHIR both within and beyond their organisations either as individuals or as designated internal representatives of PHIRIC. Founding members described themselves as active leaders and champions, promoting PHIR concepts and endeavouring to secure financial and human resources for the field: “*Our current strategic plan has population health intervention research as a core and standalone feature. It’s intertwined and almost a hub or grounding point*” (research funder); “*We are helping to steer the work. We still have commitment to it*” (research producer). They also reported an increase in awareness and support for PHIR. This is consistent with findings from a separate document review showing that the founding organisations of PHIRIC produced a much larger proportion of documents citing PHIR.^3^

Individuals and organisations that had become engaged with PHIRIC more recently, however, did not tend to define themselves as field-building leaders or champions for PHIR. They described working within the sanction and legitimacy of the collective leadership offered by PHIRIC to lead changes within their own organisations. As two interviewees noted: “*If we mean champion as a leader, that has been no. We do not have the resources. But if you mean trying to be on the cutting edge and rebalancing our pool of funds to respond, then yes*” (research funder); “*We are not champions of population health intervention research per se, but we invest in it when we can*” (research producer). According to those interviewed and at least within the PHIRIC member organisations, the profile of the PHIR field had increased over the last 5 years.

PHIRIC members also reported acting as leaders and champions of PHIR within broader national population and public health systems. A representative of one organisation noted: “*Our organization has definitely been a leader and champion both in Canada and internationally. We are asked to speak on it many times and have had many occasions to share the approach*” (research funder). Leadership and championship efforts at the system level were less apparent among organizations that had more recently joined PHIRIC, even those actively involved in carrying out or sponsoring PHIR: “*We are doing large-scale intervention research but not because of PHIRIC*” (research user); “*We have been able to show that it’s possible to do … and that governments are preoccupied [with] and willing to pay for these kinds of studies*” (research producer).

### Challenges and enablers

Interviewees reported a number of challenges and enablers in moving a PHIR agenda forward within their respective organiSations. Constraints and challenges were more frequently noted by newly engaged and former PHIRIC members compared to founding members. Challenges related to the competition with other priorities, particularly within health research funding organisations, and the difficulties in demonstrating the added value of PHIR. Enablers to advancing the PHIR agenda included the legitimacy garnered from being associated with PHIRIC and whether members felt they had the capacity and evidence to position PHIR and its value within their respective organisations.

#### Competition with other priorities

Interviewees perceived that, in the larger context of health research funding, PHIR is often a minor focus, especially in light of funding constraints. As a research funder noted, “*It’s a very competitive funding environment and it’s very difficult for population health intervention research to have a space*.”

#### Variable positioning of PHIR within member organisations

Positioning PHIR for PHIRIC’s organisational members was variable, being strong in some organisations and gaining strength in others, especially among the newer members of PHIRIC. One organisational representative stated: “*There is now greater emphasis [on population health intervention research in our organization]. It is definitely part of our mandate and goals*” (research funder). Other organisations were struggling, having achieved recognition of the importance of PHIR but not yet concrete support for it, such as targeted funding: “*We have a strong utilization in a symbolic way and a strategic way but not an operational way … There is no modality in place for [encouraging population health intervention] research that responds to the needs of decision-makers*” (research user and producer).

#### Need for evidence on value of PHIR

One of the challenges mentioned by PHIRIC members working to garner interest and overcome resistance to PHIR in their organisations and sectors was the scarcity of compelling evidence about its value. Several newer and former PHIRIC members were more likely to identify the need for more evidence from PHIR to be produced and disseminated: “*Although we are still interested, we were disappointed to see that not much was coming out of the [funded intervention] research*” (research funder); “*We need empirical evidence that [the population health intervention approach] works better in achieving change* … *We need to begin to show [whether or not] our approach works*” (research user). A key dilemma is how to show benefit from funded research if little research will be funded until benefit is shown. According to some participants, this challenge may have arisen out of an early PHIRIC decision not to limit its focus on specific public health topics and related intervention research (e.g. effectiveness of interventions to prevent childhood obesity); some respondents wondered whether concentrating research efforts on specific public health topics would have a better chance of generating early influential research success and impacts in policy and practice fields.

#### Legitimacy by association

For funding organisations, PHIRIC (and its implied stamp of approval from the primary federal government health research funder) was a key source of external legitimacy, helping to attract attention within other research funding agencies: “*It needs validation from an external source or group and that’s what PHIRIC brings.*” Participants also described PHIRIC’s role as that of a collaborator with shared interests to advance PHIR: “*Through our active involvement in PHIRIC, it’s like we’ve been on an intervention research journey together*” (research user).

It was clear from interviews that the CIHR-IPPH has played a strong role in building the field of PHIR. Moreover, its role was seen as essential to the continued advancement of PHIRIC: “*It’s important that PHIRIC stay close to [CIHR-] IPPH. I don’t see how we could work without them and we could likely fall back without their work*” (researcher). Over and above this role within PHIRIC, the CIHR-IPPH has also undertaken leadership within its parent organisation and within the health research funding community more broadly by convening meetings of major governmental and health charity funders to increase awareness in and investments for PHIR.

### PHIRIC’s individual and collective members’ influence

For many involved in PHIRIC, the initiative has a played a field-building role through enhanced awareness, profile, funding, relationships and connectivity to support PHIR, while ensuring complementarity to other related research fields in Canada. One of its important field-building benefits has been the development of shared concepts and common language about population health interventions and related research. Interviewees described PHIRIC’s impacts in part by where its language is being used, such as in the funding calls of Canadian health research funders or by international organisations: “*Internationally, the level of interest is fabulous. The language is being used broadly …. I’m really seeing that this resonates*” (researcher). For these respondents, the words are steeped in significance. Early on in its evolution, a definition for PHIR was adopted by PHIRIC, supplemented by deliberate and organised efforts to operationalise and translate the definition into concrete products such as peer-review guidelines, competencies and frequently asked questions about PHIR [28]. A supplemental issue of the 2012 *Canadian Journal of Public Health* helped further communicate the definition of PHIR and its boundaries [3]. These efforts to improve literacy in the definition, related concepts and boundaries of the field appear to have been an important step in the field-building process. However, a few former members questioned the value of PHIRIC’s focus on vocabulary and language: “*Sitting around arguing about the definitions I find completely disheartening*” (research funder).

Part of the impetus within PHIRIC to define and bound the field of PHIR seems to have been related to the concomitant building of the PHIR field or perceived encroachment of alternative fields, notably programme evaluation and implementation science, both of which are also attracting the interest of decision-makers and funders [29]. There was no consensus among current PHIRIC members regarding the relationship of PHIR to other emergent areas of study and whether PHIRIC should take steps to align PHIR with or separate itself from these fields [30]. For example, one respondent indicated a need to develop closer links to evaluation: “*Our strength is evaluating real-world interventions. We have been trying to engage in strategic positioning, for example in relationships with NGOs and government, so that we are not so disconnected from them in terms of evaluation*” (research producer). Another rejected such a linkage: “*For me, intervention research is action research, transformative research that is capable of working closely with decision-makers in a reflexive way that is very close to questions emerging from the ground. This is different in posture from evaluation research … where we do a report that is based essentially retrospectively*” (research producer).

As noted previously, PHIRIC aspired to be a strategic alliance of individuals and organisations with the overall aim of increasing the quality and quantity of PHIR. A shared, agreement-driven decision-making power, in this case among Planning Committee members, contributed to field-building. Mechanisms to codevelop mutually beneficial assets, such as the development of peer-review criteria for intervention research proposals and core competencies for training in PHIR, now being promoted to schools and programmes of public health, can also lead to field-building. Finally, administrative consolidation – in PHIRIC’s case, through the CIHR-IPPH secretariat – is a key contributing factor, while partners maintain their own independent operations and cope with changes and constraints within their own contexts [19].

Over and above these instrumental functions, interview data were striking in the way PHIRIC was consistently portrayed as a friendly convenor of 'like minds'. This was especially important to those individuals and organisations who were present from the beginning of PHIRIC and who had experienced a lack of receptivity to PHIR elsewhere: “*It has helped to feel that we are among friendly people who think the same way and thought it important to invest in this area; because it was not always the case for us*” (research funder). This welcoming space for common interests appears to have been critical to the development of new and strengthened relationships, alliances and alignment among organisations. For example, the CIHR-IPPH worked with the Public Health Agency of Canada to advance the concepts and design of the Agency’s Innovation Strategy funding programme, the first of several programmes that incorporated an explicit focus on intervention research.

Interviewees were asked to indicate to what extent changes in support of PHIR within and across organisations in Canada could be attributed to PHIRIC (i.e. to what extent PHIRIC has been successful in building the field?). Respondents agreed that the question of attribution to PHIRIC is complex. Their views varied according to their role within PHIRIC. Members who had been involved from the beginning thought strong progress had been made and expressed satisfaction with what had been accomplished*:* “*PHIRIC has been massively important as an intellectual and aspirational network. If we had not seen that spark from that network, much less would’ve been done*” (researcher); “*PHIRIC has been totally critical in moving this field forward*” (researcher). Those who had become engaged later or who had been less involved recently were not as impressed with PHIRIC’s contribution: “[*Population health intervention research] has obviously shifted but it’s not PHIRIC that has influenced it*” (research funder).

### Future prospects for PHIRIC and field-building

Expectations for PHIRIC and PHIR going forward were mixed. Longer-term and founding members of PHIRIC were more optimistic that field-building efforts will continue: “*I think it will be achieving greater traction*” (research funder); “*PHIRIC is really just getting rolling. There are many people that are extremely interested*” (researcher). Some newer and former members were equally convinced that efforts are stalled or stagnating: “*I think that population health intervention research is not even in people’s awareness …. It could be that we started at zero and are now at 10 on a scale of up to 100. We have started to see a little progress. But my perception is that not everyone knows about this*” (research producer); “*I think we are in a zone of treading water, a ceiling effect*” (research producer). These mixed reviews between long-term and founding members versus newer and past members about the ongoing need and relevance of PHIRIC raise issues about its future, at least in its current form. Despite this uncertainty, the former CIHR-IPPH Institute Advisory Board and the Propel Board of Directors both offered strong support for phase 2 of PHIRIC.

Notwithstanding diverse views on the ongoing relevance of PHIRIC, participants described a number of priority areas for building the field of PHIR. The area of greatest consensus was the need for PHIRIC to readjust its field-building bearings to support policy and practice organisations who are best positioned to use evidence from PHIR. For some PHIRIC members, field-building efforts had been about creating better conditions for researchers, which fostered their impression that PHIRIC is more of an academic think-tank and less a catalyst of research with an explicit action orientation: “*My sense is that the original vision has been lost in terms of creating a path or capacity to guide public health decisions*” (researcher). From this perspective, there was general agreement that a stronger voice from policy-makers and decision-makers is needed within PHIRIC: “*There’s an acknowledged need to build capacity on the policy side, so that policy makers can be engaged with the knowledge users around the table. There needs to be more seasoned recipients on the demand side*” (research user); “*I think it’s now time for phase two. It [PHIRIC] needs to be more of an instrument that is policy-driven by governments or ministries that make funds available to researchers*” (research producer).

The study also identified a number of constraints and challenges for the PHIR field and the initiative, ranging from competing priorities to limited evidence on the benefits and impacts such investments in PHIR were having. “*Although we are still interested, we were disappointed to see that not much was coming out of the [funded intervention] research*” (research funder); “*We need empirical evidence that [the population health intervention approach] works better in achieving change*” (research user).

## Discussion

This evaluative study provided PHIRIC members with an opportunity for critical reflection on the initiative’s role and effectiveness in providing leadership and championship to build the field of PHIR.

The main limitation of the study is that participants all had some level of engagement with PHIRIC for at least some period of time. As such, their perspectives on PHIRIC’s work in leadership and championship for the field of PHIR reflects the varied involvement and experiences of multiple organisations and sectors interested in the field over time. That said, it is not clear whether including participants who were not directly involved in PHIRIC (i.e. other research funders, provincial/territorial policy and practice organisations) would have changed the findings or whether they would have been able to comment on efforts of other organisations to advance PHIR. Also, since understanding is limited on effective leadership and championship in field-building, the analytic approach for this study was essentially inductive rather than theory based. Further evaluative studies could focus on understanding the opportunities and pitfalls of distributive leadership models in the broader context of research field-building and collective action theory.

Results showed that founding initiative members were more likely to be able to champion PHIR within their organisations and take on internal leadership roles to gain support and resources for the field of PHIR. They were strong advocates for PHIRIC as a field-developing instrument. Individual founding members continued to be enthusiastic champions for PHIR and PHIRIC, and most often attributed gains in this research domain to PHIRIC. More recent organisational members, in contrast, appeared to be less proactive; rather than leading PHIRIC, they felt legitimised or enabled to take on a championing role for PHIR. Awareness and profile of PHIR appeared to increase over this time period, also suggesting evidence of field-building. However, some members felt that without PHIRIC, the direction taken would not have been very different. The variability in these views was surprising to the EWG, notably because the PHIRIC Planning Committee had recently completed a planning process on future directions, during which this range of opinions had not surfaced.

These challenges appear to have contributed to some members disengaging from PHIRIC. Founding members, and to some extent newer members, described being active as representatives speaking for their organisations to garner support and resources for PHIR initiatives, and for PHIRIC as a legitimate actor in population health intervention research capacity development. Current members remained committed to shouldering a collective responsibility to act as leaders and champions to advance PHIRIC’s thinking and action. At the same time, it appeared that some members assumed different roles in different contexts, namely as individuals within organisations championing PHIR, as outward-facing representatives of organisations operating (or wanting to be) within the field of PHIR, and as contributors and influencers within PHIRIC. It may be of value for PHIRIC to consider how it can acknowledge and work with these multiple roles.

The results of this study confirm the early expectation that “*success in building the population health intervention field will depend heavily on purposeful alignment across organizations to enable integration of research, evaluation, surveillance, policy, and practice*” [31]. These findings also contribute to a greater understanding of field-building processes with the example of PHIR, by uncovering some of the challenges PHIRIC members have faced. Indeed, the field-building literature is relatively silent on struggles and failures in field-building in general, and on the challenges faced by alliance members in their individual and collective work as leaders and champions. Although the cost of interaction is cited as a disadvantage of participating in research field-building [10], published studies have mainly recounted success stories. Our study suggests that field-building for PHIR may threaten existing arrangements for organisational and resource allocation in applied research, as PHIRIC works to ensure that PHIR becomes valued as a legitimate field of health research, and to make space among existing (and more dominant) paradigms for setting priorities and allocating health research resources.

This aspect of field-building may require additional resources and evidence to prepare PHIRIC members to effectively address the constraints and challenges they face as organisational representatives (e.g. organisational policies dedicated to curative rather than preventive interventions [32]) alongside more deliberate member selection and terms of engagement. Future field-building efforts will also necessitate collective nimbleness to consider, analyse and respond to emerging research trends such as implementation science.

As a collaborative action initiative, the study findings are not surprising; interview data replicate previous findings about the challenges faced by emergent networks, including uneven levels of commitment among members and collaborative inertia due to what some might consider excessive attention to preparatory issues such as terminology and scope [33]. As one framework for collaborative action, collective impact may provide some useful guidance for interpreting and acting on these results. Conditions for achieving collective impacts are postulated to be a common agenda among participants based on a shared vision; consistent and shared measurement of actions and results; mutually reinforcing activities that maintain differentiation among participants, while exploring synergies at the boundaries of neighbouring mandates; a coordinated action plan; continuous and open communications; and backbone support from a separate organisation with staff and the required skill set [23, 34]. PHIRIC appears to have engineered many of these enabling conditions for achieving collective impact. However, study findings support some elements that need to be strengthened for PHIRIC to be more effective, notably a need for more compelling impact examples of PHIR success and effective communication of the value added by the field to support individual organisations’ efforts [35].

Our findings also suggest that to achieve its aspirations for the PHIR field, membership within PHIRIC should include more policy-makers and decision-makers to enhance opportunities for institutionalising PHIR results into policy decision-making processes [36]. PHIRIC may have been afflicted by the overly simplistic conceptualisation of the policy change process described by Guttman et al. [12], stemming from a researcher-push model of change. This deficiency could be overcome by engaging policy-makers and decision-makers in a tighter feedback loop with researchers and research funders [12], and by considering how research can be more fully integrated along the policy change spectrum, where such change is considered to be an iterative process and not an event [16].

Consistent with the collective impact literature, our findings support the important role of 'backbone' organisations in advancing the collective agenda, in this case the CIHR-IPPH. Functions that backbone organisations typically find challenging include building public will, advancing policy and external communications [34]. There is some evidence to suggest that the CIHR-IPPH has provided an important symbolic and legitimising role, lending a high degree of public credibility to PHIR, especially with other funding organisations and researchers. In its role as a 'backbone’ to PHIRIC, the CIHR-IPPH has particularly advanced policy related to research funding in PHIR. In addition, our data suggest that a convenor role [37] has also been important to PHIRIC’s development, supporting Gajda’s contention that, in building strategic alliances, the “*personal is as important as the procedural*” ([19], p. 69). Members described PHIRIC as a supportive environment, characterised by trust and communication (including constructive debate), which, according to the literature, is considered particularly important for achieving collective impact [23].

Overall, the results of our study support the observation that strategic alliances develop in systematic phases as they journey to effectiveness [19], with PHIRIC appearing to be on the cusp of transitioning into a new phase. Stages in strategic alliance development have been described as “*assemble and form; order and storm; norm and perform; and transform and adjourn*” [37]. Within this framework, the data collected for this study suggest that PHIRIC is somewhere between the second 'order' stage, where alliance members establish their roles and develop the initiative’s strategies and work plan, and the third 'perform' stage, where members invest in executing the work plan, moving beyond networking into cooperation or partnership levels of integration [19]. In terms of collective leadership and championship of PHIR, moving more fully into 'norm and perform' stage may require PHIRIC to operate from a more fulsome and realistic appraisal of the challenges its organisational members face in pursuing a common goal in resistant and competitive intra- and inter-organisational contexts, and in increasingly dynamic and resource-constrained research, policy and practice environments. At the same time, PHIRIC should continue to infuse a sense of shared responsibility for achieving common strategic objectives. As Parkes et al. [10] suggest, this could involve ensuring that collaborative action is regarded as the basis for joint learning across participants’ different knowledge and organisational bases and stages of engagement with PHIRIC. It could also be in line with the shared measurement element of collective impact [24], which includes discerning how the common agenda represented across members will be measured and add value, and to document that value and broader impact beyond activities being undertaken within member organisations.

## Conclusion

This study contributes to understanding field-building specifically in terms of PHIR and more generally. Findings indicate that PHIRIC mobilised some but not all of what is needed to build PHIR into a legitimate, valued, ‘normative’ research field. For builders of this and other research fields, this study suggests that multi-leveled efforts, collaborative action and distributed leadership are required but not necessarily sufficient. There is also a somewhat paradoxical need to ensure that compelling early research results are available to make the case for reallocation of scarce research funding toward the emerging field.

The study signals the need to be responsive to emerging competition and priorities such as the need for incorporating perspectives of decision-makers into PHIRIC directions and initiatives. This loosely knit alliance^4^ with shared objectives holds particular promise for understanding what enablers need to be put in place to build a field, with particular applications to population health intervention research. Lessons from this “made in Canada” model may be of interest to other countries regarding the alliance structures necessary for PHIR field-building. Future evaluative studies could focus on further understanding opportunities and pitfalls of distributive leadership models in the broader context of research field-building and collective action theory. 

## Additional file

Additional file 1: Assessment of leadership and championship of PHIR at organizational and system levels–Key informant interview guide (v4 06.01.14). (DOCX 20 kb)

## Abbreviations

CIHR-IPPH: Canadian Institutes of Health Research—Institute of Population and Public Health
EWG: Evaluation Working Group
PHIR: Population health intervention research
PHIRIC: Population Health Intervention Research Initiative for Canada

## Footnotes

1 Population Health Intervention Research Initiative for Canada (PHIRIC) Workshop Report, September 26–27, 2006, Banff Centre, Banff, AB; CIHR Internal document.

2 The Working Group is comprised of five PHIRIC members from research and research user organizations with interest and expertise in evaluation. Its Terms of Reference are found online: http://www.cihr-irsc.gc.ca/e/44584.html

3 These organizations contributed 66% of all documents to the review.

4 With the recent change in leadership for the PHIRIC secretariat in the spring of 2016 to the Propel Centre for Population. Health Impact (University of Waterloo), these findings provide timely insights to inform plans for phase 2 of the initiative. These include the need to formalize expectations of members regarding their role in championing PHIRIC and/or proactively contributing to PHIRIC through revisions to Planning Committee membership and terms of reference and other strategies. While the field has moved from occasional evaluations of small-scale intervention projects, to large-scale programs of intervention research, which include engagement of policy-makers and/or practitioners, these are not well-integrated across all levels of local, provincial/territorial and federal systems.
