# Supplementary material for: Alliance members’ roles in collective field-building: an assessment of leadership and championship within the Population Health Intervention Research Initiative for Canada
Source: Health Res Policy Syst. 2017 Dec 6;15:101. doi: 10.1186/s12961-017-0265-x (PMC5718088; doi:10.1186/s12961-017-0265-x)
Supplement: Additional file 1: — Assessment of leadership and championship of PHIR at organizational and system levels–Key informant interview guide (v4 06.01.14). (DOCX 20 kb) [file 12961_2017_265_MOESM1_ESM.docx]

**Assessment of leadership and championship of PHIR at organizational and system levels –**

**Key informant interview guide (v4 06.01.14)**

The Population Health Intervention Research Initiative for Canada (PHIRIC) aims to increase the quantity, quality and use of population health intervention research in Canada. Through PHIRIC, an alliance of individuals and organizations has been engaged, which encourages alliance members to take on leadership and championship of PHIR within their organizations and among their partners, and to inform PHIRIC’s directions through feedback from this study. This collective leadership and championship of PHIR is one of PHIRIC’s four strategic objectives and recognized as a critical success factor for the initiative.

Through an evaluative study that includes a document review and key informant interviews, PHIRIC’s Evaluation Working Group is documenting PHIRIC’s efforts and outcomes in leadership and championship of PHIR in order to inform its progress moving forward. The questions being addressed in this study are:

1. To what extent and how have PHIRIC members (current and former) undertaken a role in leading and championing change that supports the development of population health intervention research, at three levels: within their organization, within PHIRIC, and within the population and public health system more broadly?
2. What degree of influence have PHIRIC members had on the development of population health intervention research, and why?
3. What could PHIRIC do to further support the development of population health intervention research in members’ organizations, at the within PHIRIC, and more broadly?

You are being asked to participate in a key informant interview for this study, because you are current/former member of the PHIRIC Planning Committee or a Working Group. The interview will take about 45 (60 if group) minutes. Your responses will remain confidential to the evaluation consultant: the Evaluation Working Group will not be aware of which individuals and organizations did and did not participate in the study. Only aggregated data will be reported and no individual or organization, other than IPPH, will be identified in the report. The report will be made available to PHIRIC members and will be published as a research article.

Your involvement with PHIRIC may have been as an individual or as a representative of one of more organizations. In the interview, please let me know whether you are providing your view as: an individual outside of an organizational affiliation OR; as an individual from perspective informed by your position within an organization; OR on behalf of an organization (and if more than one, which one).

**Section 1 - History and evolution of PHIR**

1. How did you come to be involved in PHIRIC?
2. What specifically have you/your organization(s) contributed to PHIRIC?
3. At the time you began your involvement in PHIRIC, what was the level of activity or interest in PHIR in your organization?
4. At that time, how were population health interventions and related intervention research carried out within your organization (planned, implemented, evaluated)?
5. *(if speaking from an organizational perspective)* Since you became involved with PHIRIC, how - if at all – has the level of activity or interest in PHIR in your organization changed? How has it changed, and for what population health domains? What activities of your organization are concrete manifestations of these changes? (please provide illustrative examples)
6. *(if speaking from an organizational perspective)* How is PHIR currently positioned in your organization? – What is the level and state of formalization of organizational commitment and support to PHIR? To what extent is it in a position of strategic importance to the organization?
7. *(if speaking from an organizational perspective)* What factors have contributed to this - factors internal and external to your organization?
8. What contribution has PHIRIC made to the status and positioning of PHIR in your organization? What would have happened anyway, without PHIRIC?
9. This is PHIRIC’s definition of PHIR:

*“Population health intervention research involves the use of scientific methods to produce knowledge about policy and program interventions that operate within or outside of the health sector and have the potential to impact health at the population level*.”

Is your organization’s current understanding of PHIR consistent with this definition? How or how not (e.g., are there domains that you see as excluded)? Has this changed over time, and if so, how?

**Section 2- Leadership & championship of PHIR**

We are interested in how PHIRIC members have understood and acted on their role as leaders and champions of PHIR, at three levels: within their organizations, at the PHIRIC table, and more broadly, in the population and public health system.

1. Would you say that you/your organization has been able to act as a leader and champion of PHIR, and if so how, in what domains and arenas?
2. What degree of influence would you say PHIRIC, through you, has had on the development of PHIR in your organization? Why?
3. What degree of influence would you say your organization, or you have had on PHIRIC’s orientations? How has your role and experience within your organization shaped what you have brought to PHIRIC?
4. What other individuals, organizations or other factors (events, publications/products, developments or conditions have also influenced the PHIRIC initiative, and how?
5. To what extent and how has PHIRIC as a strategic alliance been important in developing PHIR in Canada since it was formed in 2006? If it not been present, what do you think would have happened in the evolution of PHIR over this time? How about internationally?

**Section 3: Future developments and needs (if time allows)**

1. Going forward, what do you expect will occur in your organization with respect to PHIR – holding steady, moving forward, regressing? In what ways? What factors will affect this – inside the organization and externally?
2. What aspects of PHIRIC have you found most and least valuable to date?
3. In what ways, or with what specific supports or resources, could PHIRIC support future development of PHIR: within your own organization, as strategic alliance, and in the population and public health system?

*Thank you for your collaboration!*
